# Editorial: Host-pathogen crosstalk: implications in host cellular processes by intracellular pathogens

**DOI:** 10.3389/fmicb.2024.1508345

**Published:** 2024-10-23

**Authors:** Deeksha Tripathi, Rajni Garg

**Affiliations:** ^1^Microbial Pathogenesis and Microbiome Lab, Department of Microbiology, Central University of Rajasthan, Ajmer, Rajasthan, India; ^2^Department of Human Genetics and Molecular Medicine, Amity Institute of Health Sciences, Amity University Punjab, Mohali, India

**Keywords:** host-pathogen, intracellular, small RNA, persistence, therapeutic targets

The intricate interplay between host and pathogen is fundamental aspect of microbial pathogenesis, with far-reaching implications on human health, ecological balance, and global disease burden. In this Research Topic, we delve into the multifaceted nature of host-pathogen interactions, including the morphological and metabolic diversity of bacteria, persistence and small RNA regulatory mechanisms in intracellular pathogens like, *Mycobacterium tuberculosis*. The Research Topic also includes studies on the manipulation of host microRNAs by pathogens, the interplay between microbiota and viral infections, the genetic diversity and virulence modulation of the zoonotic parasite *Toxoplasma gondii*, and the comprehensive exploration of characteristics across various intracellular pathogens. These studies highlight the critical need for enhanced research to decipher the complexities of host-pathogen interactions and develop targeted interventions that mitigate human suffering while preserving ecosystem integrity.

Diverse pathogenic characteristics and mechanisms of a wide array of microorganisms including bacteria, viruses, protozoa, fungi and helminths, have been explored by Shukla et al.. The review highlights the intricate pathogenic mechanisms employed by these organisms, emphasizing their roles in host infections. Moreover, the authors stress upon the need for further study of both harmful and beneficial microorganisms for advancing agricultural practices and public health. The dual focus on pathogenic and beneficial microorganisms aims to address health threats while leveraging their positive aspects for sustainable development (Shukla et al.).

Soni et al. discuss the morphological and metabolic diversity of bacteria, highlighting their ecological significance and implications for human health. The authors provide insights into the structure, function, and pathogenicity of bacteria based on their classification. The intricate relationship between bacterial metabolism and host interactions underscores the complexity of microbial ecology. A multifaceted interplay of factors, primarily revolving around the host's immune system, genetic predispositions, and environmental conditions, have been found to influence the host's susceptibility to bacterial infections. The authors also address the escalating challenge of antimicrobial resistance (AMR) which poses a global threat to public health. Combatting AMR requires a comprehensive strategy that integrates human, animal, and environmental health, emphasizing the importance of “One Health” approach (Soni et al.).

The intricate dynamics of bacterial persistence is particularly exemplified in *Mycobacterium tuberculosis* and *Mycobacterium smegmatis*, as demonstrated by the research conducted by Joshi et al.. This study investigates the multifactorial mechanisms underpinning antibiotic tolerance, biofilm formation, and macrophage survival through a transposon mutant library analysis of *M. smegmatis mc*^2^*155*. Their findings suggest that bacterial persistence is a complex phenomenon involving various metabolic pathways and stress responses. Various loci *msmeg_3233* (CydA), *msmeg_0719, bioB, msmeg_0392* and *msmeg_2263* (hybC) were identified to play critical roles in energy production, stress management, and survival strategies, highlighting their potential as targets for novel therapeutic interventions against persistent infections. The study underscores the necessity of further research on these genes and their orthologs in *M. tuberculosis* to develop effective treatments for chronic infections (Joshi et al.).

Small regulatory RNAs (sRNAs) in *Mycobacterium tuberculosis* (Mtb) form a complex regulatory network that is vital for the pathogen's adaptation, virulence, and survival within the host (Garg et al.). These sRNAs modulate gene expression at transcriptional, post-transcriptional, and translational levels, enabling Mtb to endure the hostile conditions within macrophages. The differential expression of key sRNAs, such as MTS2823 and DrrS, in response to stressors like hypoxia and nutrient limitation, underscores their critical role in Mtb's pathogenesis. This intricate interplay between sRNAs, transcriptional regulators, and mRNA targets highlights the complexity of Mtb's regulatory framework. Targeting specific sRNAs associated with virulence or antibiotic resistance may reveal new strategies for addressing drug-resistant tuberculosis and enhancing treatment outcomes. Furthermore, insights gained from investigating sRNA networks in Mtb could pave way for investigating similar regulatory mechanisms in other pathogens, leading to innovative approaches for combating infectious diseases (Garg et al.).

Latent tuberculosis infection (LTBI) possesses the potential to progress to active tuberculosis (ATBI), thereby facilitating the transmission of disease. This process is intricately influenced by *Mycobacterium tuberculosis* (Mtb) through its manipulation of host microRNAs (miRNAs). Exosomal miRNAs, particularly miR-155, miR-125b, and miR-29a, are crucial in modulating the immune response to tuberculosis (TB) by influencing macrophage and T cell differentiation. Their stability makes them valuable biomarkers for distinguishing active TB from latent infections. miRNA-based therapies show promise in enhancing immune responses and combating drug resistance. The role of exosomal miRNAs in TB underscores the potential for innovative diagnostic and therapeutic strategies, which could significantly improve patient outcomes and address the challenges posed by drug-resistant strains (Mukhtar et al.).

Recent studies investigating the role of transcriptionally active microbes (TAMs) in the serum of dengue-positive individuals using RNA sequencing by Yadav et al., revealed distinct microbial compositions linked to viral loads. High viral loads were found to be correlated with increased opportunistic microbes like *Campylobacter*, while low viral loads, associated with milder symptoms, showed more commensals such as *Lactobacillus*. Additionally, distinct lymphocyte and neutrophil counts were observed between the groups, suggesting that blood parameters and specific microbial patterns could serve as prognostic markers for disease progression. These findings highlight the importance of considering both microbiome and host factors in assessing dengue progression and developing interventions, offering a new perspective on combating this global health challenge (Yadav et al.).

Recent studies on *Toxoplasma gondii* have revealed intriguing strain-specific effects on host cells. This genetically diverse parasite, known for modulating host functions, shows varying impacts across its three main lineages. Notably, strains Me49 and NED induce host cell cycle arrest and chromosome missegregation, differing from the haplotype I (RH strain). Both strains increase binucleated cell formation, indicating cytokinesis failure, while NED uniquely alters cyclin B1 expression, indicating distinct host adaptations across haplotypes. These findings highlight complex host-parasite interactions and their potential influence on severity of toxoplasmosis. Such insights into strain-specific mechanisms may guide future research and inform targeted interventions in both human and veterinary medicine, addressing this significant public health concern (Rojas-Barón et al.).

Recent research has identified promising peptides that inhibit *Leishmania* invasion of host cells, a crucial step in leishmaniasis infection. Using phage display technology, Verga et al. identified two effective peptides that inhibit the interaction between metacyclic promastigotes (MPs) of Leishmania species and phagocytic host cells: La1, specific to *L. amazonensis*, and Li1, a dual-targeting peptide. Both have been found to reduce parasite internalization by 44% *in vitro*, while Li1 decreased visceral leishmaniasis infection in mice by 84%. These findings open new avenues for developing targeted treatments against this neglected tropical disease, potentially revolutionizing our approach to leishmaniasis management (Verga et al.).

Addison et al. recently investigated the role of geographic variations in the pathogenicity of *Photorhabdus asymbiotica* isolates. They found that, while European soil isolates lack mammalian cell survival, Australian and North American clinical strains selectively infect human immune cells, with infectivity dependent on growth temperature. A new clinical strain, *P. luminescens* was also found to infect human cells. These findings illuminate the role of geographic differences in development of virulence mechanisms, further expanding our understanding of pathogenic potential of this genus (Addison et al.).

These explorations of host-pathogen crosstalk offer critical insights into the pathogenic mechanisms influencing host cellular processes. As research continues to unravel the multifaceted relationships between hosts and pathogens, it becomes evident that a comprehensive approach—integrating ecological, genetic, and immunological perspectives—is essential for addressing public health challenges. By focusing on both harmful and beneficial microorganisms, we can develop sustainable practices that address health threats while harnessing the positive aspects of microbial diversity. This holistic understanding will be crucial for advancing therapeutic outcomes and improving disease management strategies in the future.

